# Development and characterization of dendritic cell internalization and activation assays contributing to the immunogenicity risk evaluation of biotherapeutics

**DOI:** 10.3389/fimmu.2024.1406804

**Published:** 2024-08-20

**Authors:** Michel Siegel, Aman Padamsey, Anna-Lena Bolender, Patrick Hargreaves, Johannes Fraidling, Axel Ducret, Katharina Hartman, Cary M. Looney, Cristina Bertinetti-Lapatki, Olivier Rohr, Timothy P. Hickling, Thomas E. Kraft, Céline Marban-Doran

**Affiliations:** ^1^ Roche Pharmaceutical Research and Early Development, Pharmaceutical Sciences, Roche Innovation Center Basel, Basel, Switzerland; ^2^ Roche Pharmaceutical Research and Early Development, Pharmaceutical Sciences, Roche Innovation Center Penzberg, Penzberg, Germany; ^3^ University of Strasbourg, UPR CNRS 9002 ARN, IUT Louis Pasteur, Schiltigheim, France; ^4^ Institut Universitaire de Technologie Louis Pasteur, Université de Strasbourg, Schiltigheim, France

**Keywords:** immunogenicity, immunomodulation, biotherapeutics, dendritic cells, assay development

## Abstract

**Introduction:**

Immunogenicity refers to the ability of a substance, such as a therapeutic drug, to elicit an immune response. While beneficial in vaccine development, undesirable immunogenicity can compromise the safety and efficacy of therapeutic proteins by inducing anti-drug antibodies (ADAs). These ADAs can reduce drug bioavailability and alter pharmacokinetics, necessitating comprehensive immunogenicity risk assessments starting at early stages of drug development. Given the complexity of immunogenicity, an integrated approach is essential, as no single assay can universally recapitulate the immune response leading to the formation of anti-drug antibodies.

**Methods:**

To better understand the Dendritic Cell (DC) contribution to immunogenicity, we developed two flow cytometry-based assays: the DC internalization assay and the DC activation assay. Monocyte-derived dendritic cells (moDCs) were generated from peripheral blood mononuclear cells (PBMCs) and differentiated over a five-day period. The internalization assay measured the accumulation rate of therapeutic antibodies within moDCs, while the activation assay assessed the expression of DC activation markers such as CD40, CD80, CD86, CD83, and DC-SIGN (CD209). To characterize these two assays further, we used a set of marketed therapeutic antibodies.

**Results:**

The study highlights that moDCs differentiated for 5 days from freshly isolated monocytes were more prone to respond to external stimuli. The internalization assay has been shown to be highly sensitive to the molecule tested, allowing the use of only 4 donors to detect small but significant differences. We also demonstrated that therapeutic antibodies were efficiently taken up by moDCs, with a strong correlation with their peptide presentation on MHC-II. On the other hand, by monitoring DC activation through a limited set of activation markers including CD40, CD83, and DC-SIGN, the DC activation assay has the potential to compare a series of compounds. These two assays provide a more comprehensive understanding of DC function in the context of immunogenicity, highlighting the importance of both internalization and activation processes in ADA development.

**Discussion:**

The DC internalization and activation assays described here address key gaps in existing immunogenicity assessment methods by providing specific and reliable measures of DC function. The assays enhance our ability to pre-clinically evaluate the immunogenic potential of biotherapeutics, thereby improving their safety and efficacy. Future work should focus on further validating these assays and integrating them into a holistic immunogenicity risk assessment framework.

## Introduction

Immunogenicity, defined here as the propensity of a substance to elicit an immune response, is a double-edged sword in the realm of biomedicine. While immunogenicity can be desirable in some contexts, such as vaccine development, undesirable immunogenicity can negatively impact the safety and efficacy of biotherapeutics. Anti-drug antibodies (ADAs) can compromise the therapeutic efficacy and safety by diminishing drug bioavailability or altering its pharmacokinetic profile. It is therefore critical to assess the immunogenic potential of biotherapeutics during their early development stages.

The complexity of immunogenicity necessitates a multifaceted assessment approach, as no single assay can universally predict the immunogenic response to protein therapeutics. This has been acknowledged by experts who recognize the limitations of current preclinical tools in forecasting clinical immunogenicity (1). A holistic strategy that interrogates various aspects of the immune system may improve the predictability of clinical outcomes and foster the development of safer, more efficacious treatments.

ADA production is triggered by a cascade of immunological events initiated by antigen (Ag) uptake by professional antigen-presenting cells (APCs), particularly dendritic cells (DCs). These cells process the internalized Ag and display peptide fragments as peptide-MHC-II (pMHC-II) complexes on their surface. T cells that recognize these complexes, along with receiving additional co-stimulatory signals, can trigger B cell activation and maturation into plasmablasts and plasma cells, which then secrete ADAs. Given the pivotal role of DCs in this process, assays such as MHC-II Associated Peptide Proteomics (MAPPs) are frequently employed in drug development to evaluate their capacity to present drug-derived peptides (2). However, other aspects of DC biology, such as antigen internalization and activation, are less explored. This is despite their importance in ADA development, recognized by studies like those by Xue et al. (3) and others focusing on protein aggregates (4, 5).

In this manuscript, we present a novel *in vitro* approach to quantify the internalization of therapeutic antibodies by monocyte-derived dendritic cells (moDCs) and to assess their subsequent activation, which is a prerequisite for an immunogenic response. Activation markers such as CD40, B7 (CD80, CD86), CD83, DC-SIGN (CD209), and HLA-DR are commonly used as indicators of the status of DC activation (6–8). Despite the challenges in detecting moDC activation by non-aggregated, monomeric antibodies, recent advancements have been made (9) building on this progress, we present novel techniques for assessing antibody internalization and DC activation, providing a more comprehensive assessment of the risk for immunogenicity. The characterization of these methods highlights the critical role of DCs in initiating immunogenic responses, one that is of high relevance in the development of biotherapeutics (10).

## Results

### Development and characterization of a DC activation assay

**Figure 1:**
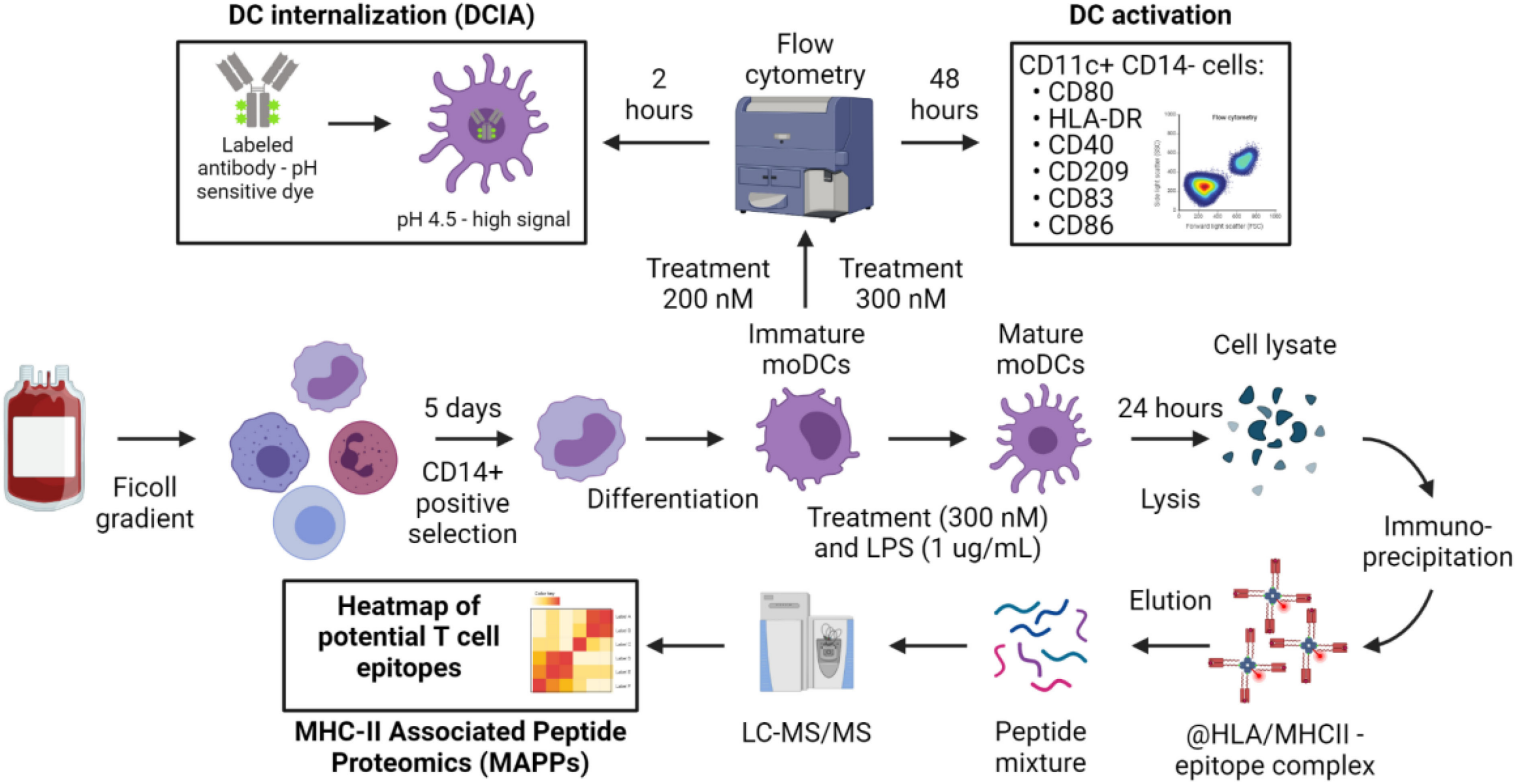
Overview of the experimental procedure to assess the contribution of dendritic cells to immunogenicity. The common starting point of the assays is the PBMC isolation according to standard protocols. CD14+ cells isolation and differentiation into immature moDCs are also shared between the procedures. Immature moDCs are challenged with the treatment as described in Material and Methods for the DC internalization and DC activation assays. Matured moDCs are used for MHC-II Associated Peptide Proteomics (MAPPs).

Activation, internalization, processing and presentation of biotherapeutics into antigen presenting cells (APCs) are the first step in the immunogenic response to protein based therapeutics. We therefore used moDCs as APCs to look into their activation status, their propensity to internalize therapeutic antibodies and to present drug-derived T cell epitopes. The workflow of the three immunogenicity assays used in the present manuscript are depicted in **Figure 1**.

**Figure 2:**
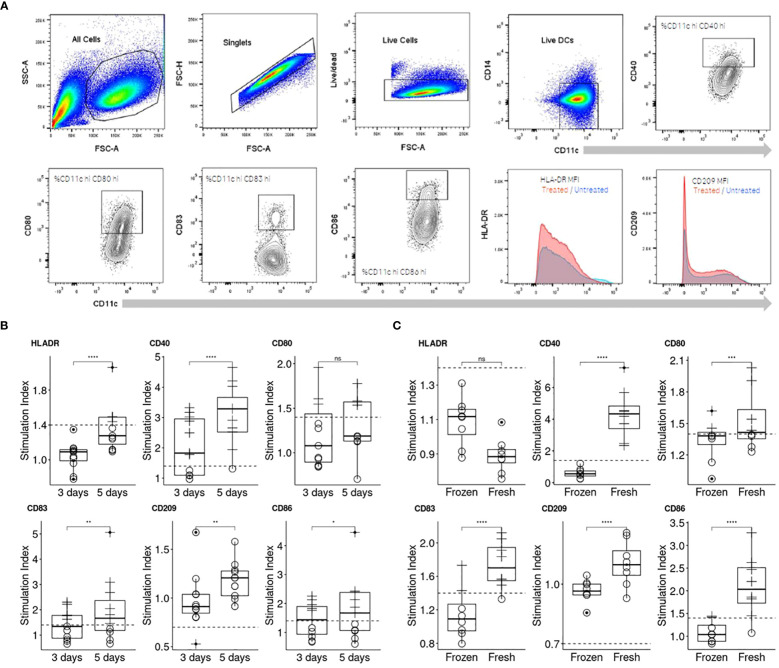
Comparison of two moDC differentiation durations and cell sources on their response to KLH. **(A)** Representation of the flow cytometry gating strategy applied for the assessment of DC activation. **(B)** Three days of differentiation was compared to an extended differentiation of five days by assessing the moDC response to KLH (n=10). Individual moDCs SI were calculated (see Material and Methods, “Data analysis” section for more information) and a plot per activation marker generated. **(C)** Freshly isolated and frozen PBMCs were compared for their ability to respond to KLH (n=8). To compare the differentiation periods and the PBMC source, one-sided paired t test for each activation marker were performed (p< 0.0001****; p< 0.001***; p< 0.01**; p< 0.05*; not significant, ns).

Numerous protocols for differentiating monocytes into moDCs *in vitro* have been documented in the literature (11). Drawing from our experience and the internal use of moDCs in the MAPPs assay (2), we chose a five-day differentiation period as our initial approach for cell generation, as it has been published that this timeframe is sufficient to differenciate CD14+ cells into moDCs. To ensure optimal moDC phenotype on the day of antigen challenge with the antibody, we compared this differentiation protocol to a shorter version of 3 days published recently (9). The assessment was conducted using flow cytometry, as detailed in the Materials and Methods section. In summary, cells were selected based on singlets, morphology, and viability. We then measured the Mean Fluorescence Intensities (MFI) for HLA-DR and CD209 and the percentage of positive cells for other activation markers (CD80, CD86, CD83, and CD40) on viable CD11c+ CD14- cells (**Figure 2A**).

The assay was initially evaluated for its sensitivity to lipopolysaccharide (LPS) treatment, which is known to activate moDCs through the TLR-4 signaling pathway (**Supplementary Figure 1A**). The panel of activation markers we examined (HLA-DR, CD40, CD86, CD83, CD209, and CD80) exhibited similar responses under both differentiation protocols (3 versus 5 days). Notably, all markers were upregulated in a dose-dependent manner, with the exception of CD209, which was downregulated as anticipated (**Supplementary Figure 1B**). However, since LPS induces activation of moDCs through receptor-mediated pathways, we also tested keyhole limpet hemocyanin (KLH), an antigen that does not engage with specific surface receptors on moDCs (**Figure 2B**) and is expected to therefore activate comparable pathways to those activated by drug internalization. The moDC response to KLH was less pronounced than to LPS, allowing us to more clearly discern the nuances in response dynamics. moDCs demonstrated an upregulation of all markers to KLH following 5 day period of differentiation, with the sole exception being the downregulation of CD209. Another critical factor was the origin of the monocytes. We observed significant differences in moDC responsiveness to KLH when comparing moDCs derived from freshly isolated peripheral blood mononuclear cells (PBMCs) with those derived from frozen PBMCs (**Figure 2C**). Indeed, the median SI for CD40 increased approximately by 4-fold while for CD80, CD83, CD86 it increased by 1.1, 1.3 and 2 respectively. Consequently, we recommend using freshly isolated PBMCs and employing the response to KLH as a positive control to verify cell viability and functionality.

**Figure 3:**
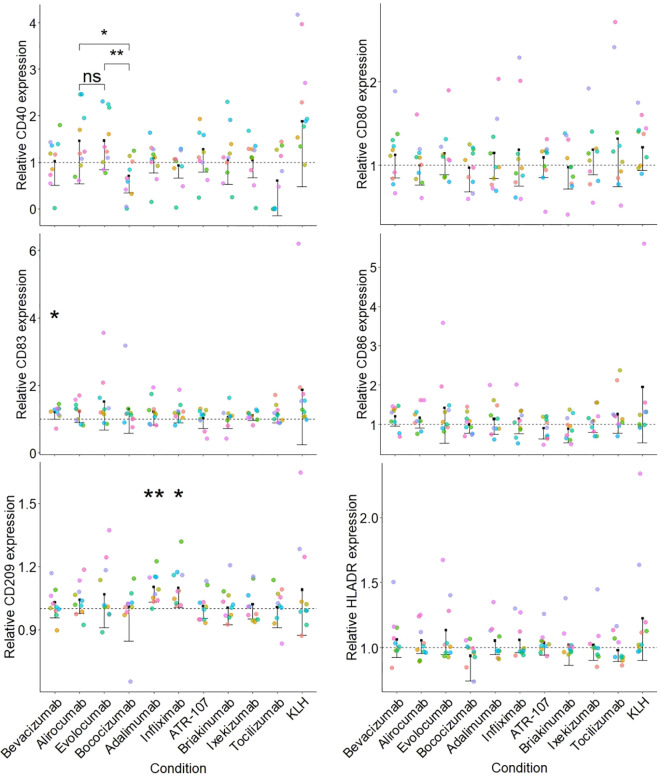
Activation of moDCs by a set of therapeutic antibodies. Each sub-figure represents the stimulation index (SI) for a particular surface marker and the dotted line corresponds to 1 (value for the medium treated condition). The SI was calculated for each donor/surface marker pair using the corresponding medium treated control as described in the Material and Methods section. Each individual donor tested is indicated by a different color (n=10) and a one-sided paired t test was applied for the comparison of antibodies against the medium treated control (p< 0.01**; p< 0.05*) displayed along with a one-sided confidence interval (level = 0.95). Additionally, a paired one-sided t test between the PCSK9 targeting antibodies (alirocumab, evolocumab and bococizumab) was performed (p< 0.01**; p< 0.05*; not significant, ns).

The parameters for the assay as mentioned above were used to qualify the DC activation assay using a set of commercially available therapeutic antibodies (**Figure 3**).

None of the tested therapeutic antibodies significantly affected the phenotype of the moDC, with the exception of CD209 expression, which was increased (15% increase above medium-treated control) by the TNFɑ targeted antibodies adalimumab and infliximab. We observed a similar, albeit non-significant (when compared to the medium treated control) increase in CD40 expression following treatment with the PCSK9 targeting antibodies alirocumab and evolocumab, but not for bococizumab. Of note, this observation might be a consequence of a decreased expression of CD40 following bococizumab challenge.

### Development and characterization of a DC internalization assay

In addition to providing a costimulation signal to T cells, APCs should ensure the specificity of the response by presenting an epitope derived from the antigen, which starts with its internalization into APCs. Measurement of the cellular accumulation of drug candidates in a meaningful way during preclinical development of therapeutic antibodies could therefore improve the understanding of their immunogenicity risk and aid in the selection and engineering of a clinical lead molecule. We therefore developed an assay to measure the internalization and accumulation rate of mAbs in human moDCs. We first labeled antibodies with a pH-sensitive fluorophore, site directed to their Fc glycosylation; this avoids the alteration of biophysical and target binding properties, as no amino acid is modified. We then incubated these labeled mAbs with moDCs and determined the relative amount of accumulated antibody through FACS measurement and normalization to dosing solution fluorescence. The fluorophore shows low to no fluorescence at physiological pH outside the cell and an 50-100 fold increase in fluorescence intensity at acidic pH, found in the late endosome and lysosome (details can be found in **Figure 1** and in the material and methods section together with the Equations 1, 2).

A set of 8 commercially available therapeutic antibodies comprised of ixekizumab, alirocumab, evolocumab, bevacizumab, briakinumab, adalimumab, bococizumab, and ATR-107, as well as two additional internal control mAbs (var1 and var112, with IgG typical and high internalization rates respectively) (12), were tested to evaluate the performance of the DCIA and to better understand properties that could influence internalization.

ATR-107 and bococizumab showed significantly higher DC internalization as compared to var1, the internal control. This was also observed to a lesser extent for adalimumab and briakinumab. The other benchmark molecules (alirocumab, evolocumab and bevacizumab) were internalized at a similar rate as the internal control, while ixekizumab showed a significantly lower internalization rate.

**Figure 4:**
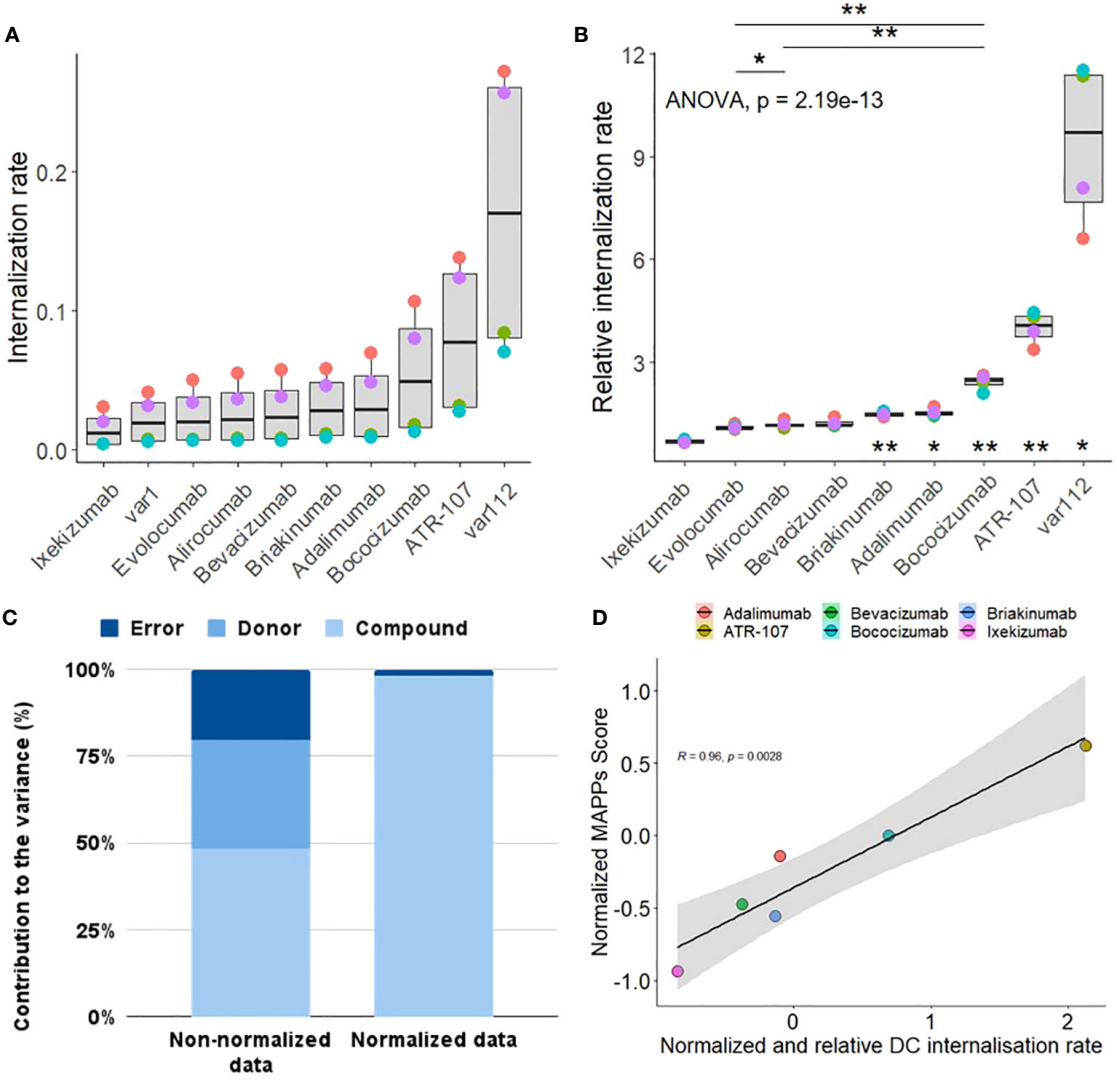
Characterization Qualification of DC internalization assay. **(A)** The internalization rate represents the internalization efficiency as calculated by the slope of the mean fluorescence intensity (MFI) values of antibodies coupled to a pH sensitive fluorophore into the acidic lysosome of CD11c+ moDCs from 4 human healthy blood donors (color coded) at 120 min, normalized to the fluorescence of the antibody dosing solution to account for differences in labeling efficiency between antibodies. **(B)** The relative internalization rate uses the slope of an internal control antibody to normalize the donor specific internalization rate (according to the Material and Methods section). A one-sided paired t-test was applied for the comparison of antibodies sharing the same target (displayed at the top, p< 0.01**; p< 0.05*). A one sample two-sided paired T-test between each group (antibody) and the internal control antibody has been performed (corrected for multiple testing, displayed at the bottom, p< 0.01**; p< 0.05*). **(C)** Comparison of the contribution of donor, compound and residual error on the total variance for the non-normalized **(A)** vs normalized internalization data **(B)**. **(D)** Correlation plot between the normalized MAPPs score (see Material and Methods for the equation used and **Supplementary Figure 3** for the heatmap of the detected peptide clusters) and the normalized and relative (to var1) DC internalization rate. Normalization for each treatment has been achieved by subtracting the corresponding assay dataset mean and dividing it by the corresponding standard deviation.

The rate of compound internalization, as calculated by dividing the mean fluorescent intensity by the incubation time, varied by donor as well as between the different compounds (**Figure 4A**). Since our goal was to capture compound specific differences in internalization in order to rank drug candidates within a series and to flag high internalization molecules, the inter-donor variability was controlled for by normalizing the slope of each test antibody for each donor with the slope of our internal control var1 (untargeted IgG1) measured in the same donor, thus yielding relative internalization rates (**Figure 4B**). On performing a Variance Component Analysis, it was shown that this method of normalization almost completely accounted for donor specific variance (**Figure 4C** and **Supplementary Table 1**). The normalization procedure reduces donor based variance from ~31% to 0%, suggesting that the residual variance was compound-specific. This inter-donor baseline correction also allows us to detect smaller differences between compounds, with high statistical significance.

For routine use of the DCIA, we aim to reduce the number of donors to optimize throughput, save resources, and decrease time and cost. This reduction still enables the detection of large enough differences between compounds to be useful in informing decisions regarding immunogenicity risk and compound ranking, as immunogenicity risk is only one of several factors defining a successful clinical lead molecule, and internalization is only one of several contributing factors for immunogenicity risk (12). We therefore decided on a minimum effect size of 2, based on internal experience with the assay, to be able to capture large, relevant differences between internalization rates of compounds and used a power analysis to determine a suitable sample size of 4 donors (**Supplementary Figure 2**).

Additionally, we explored the relevance of the observations made about the cellular accumulation rates for the therapeutic antibodies tested by comparing it to the outcome of a well-established assay, the MHC-II Associated Peptide Proteomics (MAPPs, **Supplementary Figure 3**). The set of therapeutic antibodies tested, in both the DCIA and MAPPs, exhibited a wide range of risk for immunogenicity. Interestingly, our results revealed a linear correlation between the cellular accumulation of these antibodies and their presentation by Major Histocompatibility Complex class II (MHC-II) molecules (**Figure 4D**). This correlation suggests that the extent to which an antibody accumulates within cells may be predictive of its ability to be processed and presented as peptides on MHC-II, a key step in the activation of the adaptive immune response.

## Discussion

The studies presented here focus on enhancing our understanding of the mechanisms underlying clinical immunogenicity in response to therapeutic antibodies. This can be achieved by focusing on the early stages of the immunogenic response that leads to the development of anti-drug antibodies (ADAs), specifically the internalization and presentation of antigens by dendritic cells and their activation.

The differentiation of moDCs was found to be critically dependent on both the presence and incubation conditions of IL-4, as previously reported (11). This finding reinforces the importance of standardizing differentiation protocols to ensure reproducibility and reliability in moDC-based assays. The use of serum-free media was a key factor in minimizing assay variability, given the complex and potentially variable composition of serum, and the potential uptake of serum proteins by moDCs that could interfere with the assay, as noted by Sauter et al. (13). Furthermore, our choice of ultra-low binding surfaces was based both on past experience and literature, as their use did not impair the T cell activation capacity of moDCs (13), suggesting that these surfaces are suitable for culturing cells in the context of our assays. The exclusion of LPS as a co-treatment was based on the understanding that DC maturation and/or activation could inhibit macropinocytosis, possibly confounding assay results (14, 15). This decision highlights the need to carefully consider the addition of maturation agents in assays designed to measure antigen uptake and processing.

Using the optimized protocol for moDCs differentiation, we propose an improved method for the dendritic cell (DC) activation assay with increased specificity and applicability. Here, we evaluated the ability of different therapeutic antibodies to modulate the expression of various activation markers on moDCs. Our data suggest that CD209 and CD40 are the most informative markers for assessing DC activation and should be prioritized in the assay panel. Additionally, while HLA-DR expression is more reflective of inter-donor variability than a direct response to treatment, its inclusion may remain important for capturing individual immune response differences. To streamline the assay and reduce redundancy, we recommend selecting a single co-stimulatory molecule—either CD83, CD86, or CD80—as all three have shown similar degrees of activation in the assay. For assay characterization qualification and consistency, Keyhole Limpet Hemocyanin (KLH) should be incorporated as a positive control, given its greater relevance to the tested mechanisms versus a receptor-mediated activation agent such as LPS. Finally, the inclusion of relevant comparator (e.g. sequence variants, antibody with the same target allows for a more meaningful analysis of DC activation potential.

The results of our study underscore the complex interplay between cell culture conditions, antibody characteristics, and assay protocols in influencing the phenotype and function of moDCs, with direct implications for testing the *in vivo* immunogenicity potential of therapeutic antibodies.

In addition to optimized cell culture conditions, the right assay setup and optimal controls are crucial for generating meaningful data, as shown by the use of internal antibody controls. By normalizing the cellular accumulation rate of each test compound to the rate of the negative control, we substantially reduced the donor contribution to the total variance, highlighting compound-specific differences. This is particularly important when comparing compounds.

Cellular accumulation in APCs is only one of several mechanisms that potentially contributes to the risk of immunogenicity for a therapeutic antibody. Furthermore, other parameters, like potency, pharmacokinetics and technical developability also need to be taken into account when selecting a suitable clinical lead. Therefore, the ability to measure significantly large differences between compounds regarding their DC cellular accumulation rate can be seen as a key first step for clinical lead selection.

The number of healthy donor samples required for investigating DC internalization was determined by the calculation of the effect size according to the sample size. The effect size, which indicates practical significance, was used instead of relying solely on statistical significance (p-values), which can be misleading due to its dependence on sample size. An effect size value of 2 (Cohen’s D factor >0.8 considered as large), was observed with a sample size of 4 healthy donors. This suggests that 4 samples are sufficient to detect a meaningful difference in DC internalization rates using a paired t-test. The results of a power analysis in combination with the reduction in donor and residual error by normalization enabled us to increase the throughput and number of test compounds per assay run compared to other preclinical immunogenicity assays requiring between 13-30 donors (16, 17). In addition, we recommend an internal negative control for normalization; while we used var1 in this study, bevacizumab would be a potential alternative, given its low cellular accumulation in the DCIA. Similarly, while we used var112 as a positive control, bococizumab or ATR-107 could be used as a positive control, according to our results.

Antibody characteristics such as surface charges, FcRn binding, and glycosylation patterns were shown to have significant effects on the internalization and activation of moDCs (18, 19). The increased internalization of bococizumab, which exhibits a positive surface charge patch (20) and the occurrence of ADAs in 50% of patients within a year of treatment (21) compared to other PCSK9 targeting antibodies, suggests that charge interactions may enhance uptake by moDCs and potentially increase the risk of immunogenicity. The role of target binding in antibody uptake was exemplified by the TNF-ɑ targeting antibody adalimumab, which showed increased cellular accumulation, potentially leading to DC activation and increased immunogenicity (22, 23). The TNFɑ-targeting antibodies adalimumab and infliximab also showed an increased expression of C-type lectin receptors (CD209/DC-SIGN) on moDCs, a molecule associated with increased presentation on DCs. Furthermore, this assay enables the comparison of molecules sharing the same target such as evolocumab, alirocumab and bococizumab, all three targeting PCSK9. The reduced expression of CD40 observed after treatment with bococizumab, compared to evolocumab and alirocumab, could result from a less mature phenotype of the moDCs. This may be a consequence of the higher cellular accumulation of bococizumab compared to evolocumab and alirocumab, and potentially account for a more efficient internalization into DCs.

The impact of antibody formulation on DC uptake and activation by such antibody-independent considerations as aggregation and their subsequent effect on immunogenicity also must be considered. Aggregates can profoundly influence antibody internalization and moDC activation, as demonstrated by the historical use of DC activation assays to describe the immunogenicity of antibody aggregates (24). This is particularly relevant to bococizumab and ATR-107, which were not evaluated in this context with their clinical formulation and concentration.

Immunogenicity is influenced by a multitude of factors, one of which is the efficacy of synapse formation between T lymphocytes and DCs. This interaction is enhanced when DCs display a high density of peptide epitopes on their surface, increasing the avidity of TCR/MHC interactions (25). Considering the observed positive correlation between the DCIA and MAPPs data, an increased internalization of antigens by DCs may lead to a more abundant presentation of epitopes, potentially indicating a higher risk of immunogenicity.

The production of ADAs is influenced by a wide range of risk factors, including those related to the product, the treatment regimen, and the patient population, thus making immunogenicity risk assessment complex and challenging. On the one hand, product-related risks such as the sequence or the biophysical properties of biotherapeutics are often evaluated early in the development, employing in silico and *in vitro* tools to guide candidate selection. This often involves predicting T cell epitopes via in silico tools, in parallel with conducting MHC-II Associated Peptide Proteomics (MAPPs and T cell activation assays (2, 17). On the other hand, we present the DCIA and DC activation assays to provide a more mechanistic understanding of immunogenicity. Gaining a deeper understanding of the mechanisms driving immunogenicity is critical to minimize immunogenic potential of candidates and ensure the delivery of safe biotherapeutics to patients.

## Materials and methods

### Compounds

Stock solutions of keyhole limpet hemocyanin (KLH-Imject Maleimide-Activated mcKLH, Thermo Fisher Scientific, #77600) were reconstituted and stored at -80°C in single-use aliquots according to the manufacturer’s recommendations under sterile conditions. All biotherapeutics; were either produced internally (briakinumab, ixekizumab, bococizumab, ATR-107, var1 and var112) or bought from Runge Pharma GmbH & Co in their respective formulation and stored according to the manufacturer’s recommendations.

### Antibody labeling

For the DC internalization assay, antibodies were labeled using the SiteClick Antibody Azido Modification Kit (Thermo Fisher, #S20026) according to the manufacturer’s instructions. Briefly, N-linked galactose residues of the Fc region were removed by β-galactosidase and replaced by an azide-containing galactose via the β-1,4-galactosyltransferase. This azide modification enables a copper-free conjugation of sDIBO-modified dyes. The pH-sensitive amine-reactive dye was coupled to a sulfo-DBCO PEG4 amine. Antibodies were labeled with a molar dye excess of 3.5. Excess dye was removed using the Amicon Ultra-2 Centrifugal Filter (Merck, #UFC205024) with a MWCO of 50 kD and antibodies were re-buffered in 20 mM histidine 140 mM NaCl buffer (pH 5.5). The fluorescence of the dosing solution was measured in a Tecan Infinite Pro 300 fluorometer. 50μl of dosing solution was mixed with 150μl citric acid buffer (0.2 M Citrate-Phosphate buffer pH 4.5) and the fluorescence was measured with an excitation at 532 nm and emission at 560 nm. The absorbances of the labeled molecules at 280 nm and 532 nm were determined using a Nanodrop spectrometer and the concentration [1] as well as the dye-to-antibody ratio (DAR) [2] was calculated as follows.


(1) $$\text{c}\left(\text{AB}\right)=[\text{A}280\text{nm}-\text{A}[280\text{n}{\text{m}}^{*}\;\text{CF}(\text{Dye})]]\;/\;\text{ϵ}\left(\text{AB}\right)$$

(2) $$\text{DAR}=[\text{A}532\text{n}{\text{m}}^{*}\text{MW}(\text{AB})]\;/\;[\text{c}{(\text{AB})\;}^{*}\text{ϵ}(\text{Dye})]$$

(A, absorbance; AB, antibody; c, concentration; DAR, dye to antibody ratio; ϵ (dye), extinction coefficient dye, 47225; CF, correction factor = 0.36)

### Quality control of the labeled antibodies

To confirm the efficient removal of unbound dye and to exclude possible antibody aggregates or fragments, a size exclusion chromatography of the labeled antibodies and their unlabeled counterparts was performed. Samples were separated using a BioSuite Diol (OH) column (Waters, 186002165) with a potassium dihydrogen phosphate buffer (pH 6.2) as the mobile phase at a flow rate of 0.5 ml/min. Detectors at 280 nm and 532 nm were used to quantify and analyze the labeled antibodies.

### Cell culture and maintenance

Human peripheral blood mononuclear cells were isolated by Pancoll density gradient centrifugation from whole blood according to the manufacturer’s instructions. Therefore, EDTA-whole blood donations from healthy volunteers were diluted 1:2 with PBS. For each experiment, different donors were used. For further enrichment of monocytes, magnetic activated cell sorting was performed using anti-huCD14 beads (Miltenyi, #130-050-201) and LS columns (Miltenyi, #130-042-401) according to the manufacturer’s instructions. Briefly, monocytes and beads were incubated in MACS Buffer for 15 min on ice and separated by a magnet. The isolated monocytes were suspended in a pre-warmed medium.

### Internalization assay

CD14+ monocytes were differentiated into monocyte derived DCs (moDCs), by culturing within a DC medium (sterile filtered CellGenix GMP DC medium, with GlutaMAX, non-essential amino acids, sodium pyruvate and Penicillin-Streptomycin) supplemented with 5 ng/mL rhIL4 (R&D systems, #204-IL) and 50 ng/mL rhGM-CSF (R&D system, #215GM-500) for 5 days at 37°C and 5% CO2 ambient on ultra-low attachment culture dishes (0.3x10^6^ cells/ml, Corning, #354407). On the day of the experiment, cells were detached from the ultra-low attachment culture dishes by pipetting and plated into ultra-low attachment 96-well plates at a density of 8x104 cells/well (50µl/well). Antibody solutions were prepared at a concentration of 400 nM in DC medium (dosing solution) and 50 µl were applied to the cells for a final concentration of 200 nM. Cells were incubated for two and four hours at 37°C and 5% CO2. Cells were transferred into U-bottom 96-well plates for sedimentation (300 g, 5 min), the pellet was washed with 200 µl ice cold PBS, centrifuged and resuspended in 200 µl FACS buffer containing 50 ng/mL DAPI.

### MHC-II associated peptide proteomics

MAPPs assay was performed according to the standard protocol and analyzed according to Steiner et al. (2). In short, 2.5 million moDCs (at 0.3 x 106 cells/mL) cells were challenged with the test protein at 300 nM in the presence of 1 μg/mL of lipopolysaccharide (LPS) from Salmonella abortus equi (Sigma-Aldrich Chemie GmbH, Buchs, Switzerland) for 24 h. Mature moDCs were harvested, washed with PBS and the cell pellets were frozen at −80°C. Frozen cell pellets were lysed in 20 mM Tris-buffer solution pH 7.8 containing 1% (v/v) Digitonin and protease inhibitors (Roche Diagnostics GmbH, Mannheim, Germany) for 1 h at 4°C on a ThermoMixer at 1100 rpm. The HLA-DR immune complexes were isolated by immunoprecipitation using the biotin-conjugated anti-human HLA-DR monoclonal antibodies (10 µg, clone L243, BioLegend) in a total volume of 50 µL lysis buffer (described above) per sample. Lysates were incubated with the antibody on a rotator overnight at 4°C. Samples were washed five times with a buffer containing 20 mM N-(2-hydroxyethyl)piperazine-N′-ethanesulfonic acid-NaOH (pH 7.9), 150 mM KCl, 1 mM MgCl2, 0.2 mM CaCl2, 0.2 mM ethylenediaminetetraacetate, 10% (v/v) glycerol, and 0.1% (v/v) Digitonin and five times with purified water. MHC-II peptides were eluted twice from HLA-DR molecules by adding 18 μL of 0.1% trifluoroacetic acid. The eluates were collected and analyzed by tandem mass spectrometry. Detected peptides were grouped into clusters and represented along the sequence of the corresponding antibody. A numerical estimation of the MAPPs assay outcome was calculated using the number of epitopes detected and their signal intensities like follows:


$${\text{n}}_{\text{epitopes}}\times \text{ mean}\left(\text{log}2\left(\text{signal}\right)\right)$$

### DC activation assay

CD14+ monocytes were differentiated into dendritic cells (DCs), by culturing within medium supplemented with 10 ng/mL rhIL4 (R&D systems, #204-IL) and 100 ng/mL rhGM-CSF (R&D system, #215GM-500) for 5 days at 37°C and 5% CO_2_ ambient on ultra-low attachment 96-well culture plates (200 μL, 3x10^6^ cells/ml, Corning, #3262). At day 5 the cells are seeded (300 g, 5 min) and half of the medium was changed for the treatment of interest containing medium (100 μL at 600 nMol/L or 100 μg/mL for a final concentration of 300 nMol/L or 50 μg/mL) and incubated for 48 hours at 37°C and 5% CO_2_ ambient.

The cells were then spun down (300 g, 5 min) and resuspended in 200 μL PBS containing a Fixable Viability Stain BV510 (BD, #564406) and a FcR blocking agent (Miltenyi, #130-059-901) for 15 minutes at room temperature. The medium was changed for the antibody mastermix composed of CD80 BUV 737 (clone L307, BD, #741865), HLA-DR FITC (clone G46-6, BD, #555811), CD40 BV786 (clone 5C3, BD, #740985), CD209 BV421 (clone DCN46, BD, #564127), CD11c BUV395 (clone B-ly6, BD, #563787), CD14 PerCP (clone M5E2, BioLegend, #301848), CD83 APC (clone HB15E, BD, #551073), CD86 PE (clone 2331, BD, #555658) in a brilliant stain buffer (BD, #566349) - PBS solution and incubated 30 minutes at 4°C. Cells were finally washed twice in FACS buffer and the fluorescence was acquired using the Fortessa X20 (BD).

### Data analysis

The mean fluorescent intensity (MFI) of the internalized antibodies was acquired using a Fortessa X20 flow cytometer (BD) equipped with a 532 nm emitting laser. Signals were collected at 572 nm ± 35 nm. The exact same conditions, gains, and gates were used for all time points. Data extraction was performed using the FlowJo-V10.8.1 software (BD Life Sciences). Cells were gated for singlets, morphology and viability. Values of the negative control were subtracted from all geo-mean values, followed by normalization to the fluorescence intensity of the dosing solution and to our internal untargeted IgG1 control. The normalized geo-mean values from each antibody were plotted as a linear regression using R Statistical Software (v4.1.2; 26) to extract the slope (Geo Mean MFI/min for 120 min).

Concerning the activation assay, data extraction was performed using the FloJo_V10 software as well. Cells were gated for singlets, morphology and viability. MFI were extracted for CD209 and HLA-DR while % positive were used for the other activation markers (CD80, CD86, CD83 and CD40) on CD11c^+^ CD14^-^ viable cells. Values of the non-treated control were used to calculate the Stimulation Index (SI) specific to each activation marker and individual. The SI were plotted for each treatment to compare for their ability to activate moDCs. Keyhole Limpet Hemocyanin (KLH, Sigma, #SRP6195) response was used as a positive control. Statistical significance of differences in internalization rates and DC activation SI were calculated by a paired T-test. Statistical analysis was performed using R (v4.1.2; 26). Significance level: p< 0.0001= ****; p< 0.001= ***; p< 0.01=**; p< 0.05= *; not significant= ns.

## Data Availability

The raw and processed mass spectrometric data have been deposited to the PRIDE archive (https:proteomecentral.proteomexchange.org/cgi/GetDataset?ID=PXD054823) via the Mas-sIVE partner repository (MassIVE dataset identifier: MSV000095578).

## Supplementary material

Supplementary Figure 1 Comparison of two moDCs differentiation durations using LPS. **(A)** Three days of differentiation was compared to an extended differentiation of five days by assessing the moDCs response to 1 ug/mL of LPS (n=10). **(B)** A dose response to increasing LPS concentrations using the 5 days differentiation period (n=10). Individual moDCs SI for the different LPS concentrations were calculated and a plot per activation marker generated (see Material and Methods sections “DC activation assay” and “Data Analysis”).

Supplementary Figure 2 Sample size estimation for the DC internalization assay. An *a priori* power analysis was performed using R (27) to estimate sample size for a paired t-test. Based on experience, we set an effect size of 2 to be able to capture large, relevant differences between internalization rates of compounds, along with a power of 80% and p of 0.05. The power curve indicated that at least 4 donors were required to capture effect sizes of this magnitude.

Supplementary Figure 3 Heatmaps depicting the cluster profile of MAPPs-identified peptides. The sequence regions are organized according to the antibody domains (i.e., variable domain of the heavy chain (VH), constant domain of the heavy chain (CH1), variable domain of the light chain (VL), constant region of the kappa-type light chain (Ck), and the fragment crystallizable (Fc) region). Vertical pink, green, and blue lines along the sequence of the VH and VL domains correspond to the position of the complementarity-determining regions (CDRs) 1 to 3. Identified peptide clusters are depicted as colored regions with varying abundances (as a log score) per sequence position, spanning from dark red to yellow. Donor number is denoted on the vertical axis.

Supplementary Table 1 Variance Component Analysis (28) comparing the origins of variance in non-normalized and normalized data. * VC coefficient for “Donor” assigned 0 (shrinkage) VC (Variance Component) denotes extent of the variance emanating from the corresponding variable. Error denotes residual error of the fit.
