# Supplementary figures and images for: Development and characterization of dendritic cell internalization and activation assays contributing to the immunogenicity risk evaluation of biotherapeutics
Source: Front Immunol. 2024 Aug 20;15:1406804. doi: 10.3389/fimmu.2024.1406804 (PMC11368763; doi:10.3389/fimmu.2024.1406804)

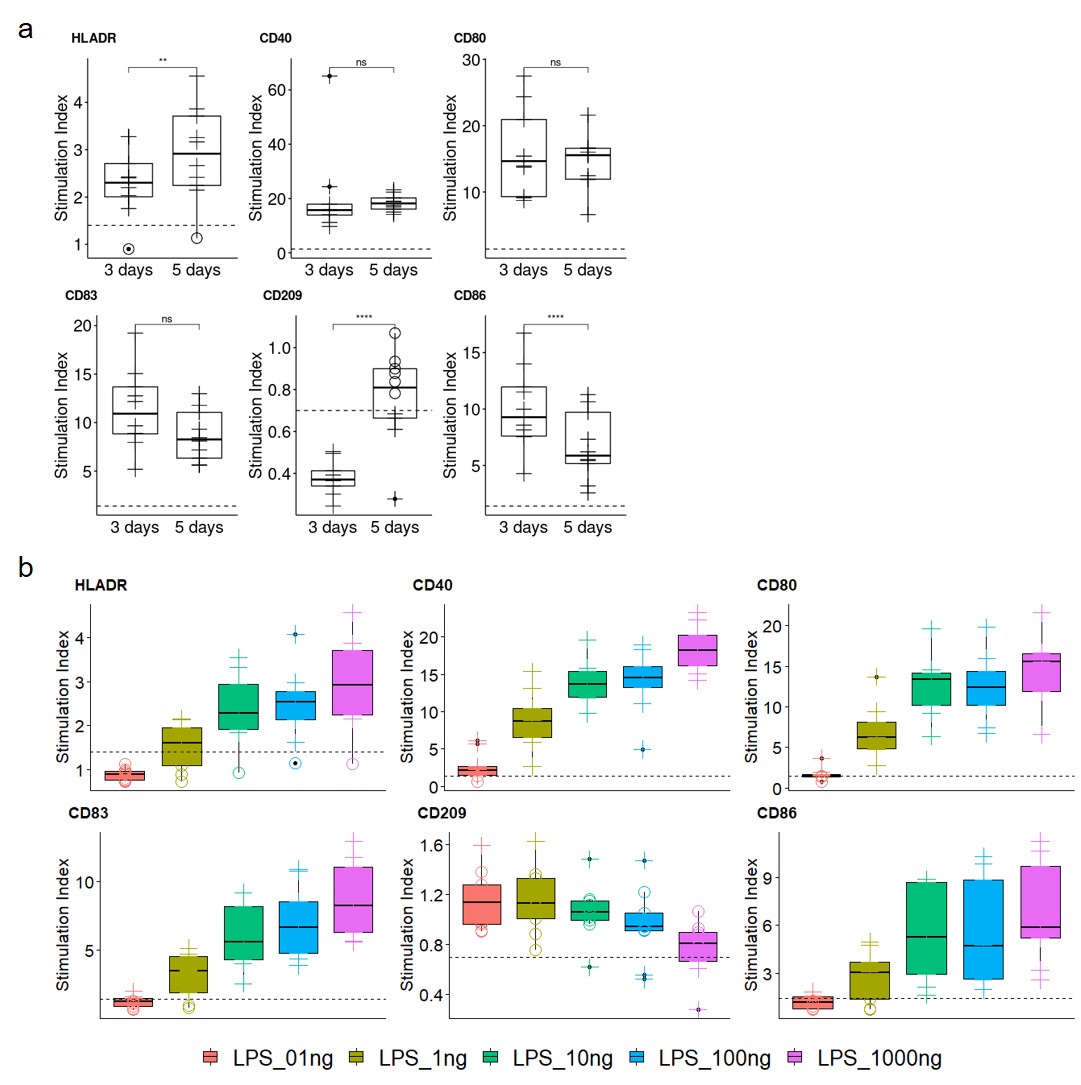

Supplement: Supplementary Figure 1 — Comparison of two moDCs differentiation durations using LPS. (A) Three days of differentiation was compared to an extended differentiation of five days by assessing the moDCs response to 1 ug/mL of LPS (n=10). (B) A dose response to increasing LPS concentrations using the 5 days differentiation period (n=10). Individual moDCs SI for the different LPS concentrations were calculated and a plot per activation marker generated (see Material and Methods sections “DC activation assay” and “Data Analysis”). [file Image1.jpeg]

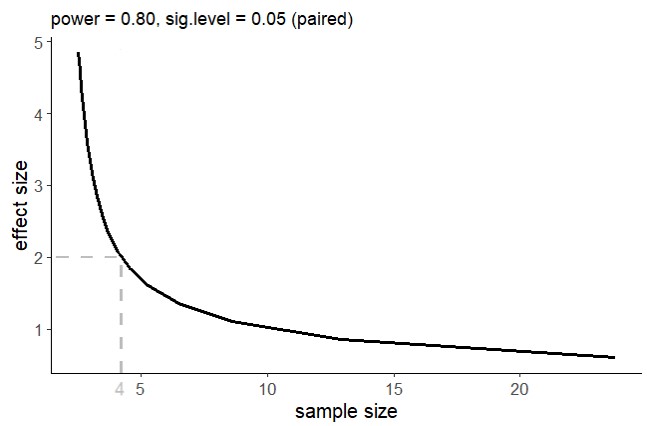

Supplement: Supplementary Figure 2 — Sample size estimation for the DC internalization assay. An a priori power analysis was performed using R (27) to estimate sample size for a paired t-test. Based on experience, we set an effect size of 2 to be able to capture large, relevant differences between internalization rates of compounds, along with a power of 80% and p of 0.05. The power curve indicated that at least 4 donors were required to capture effect sizes of this magnitude. [file Image2.jpeg]

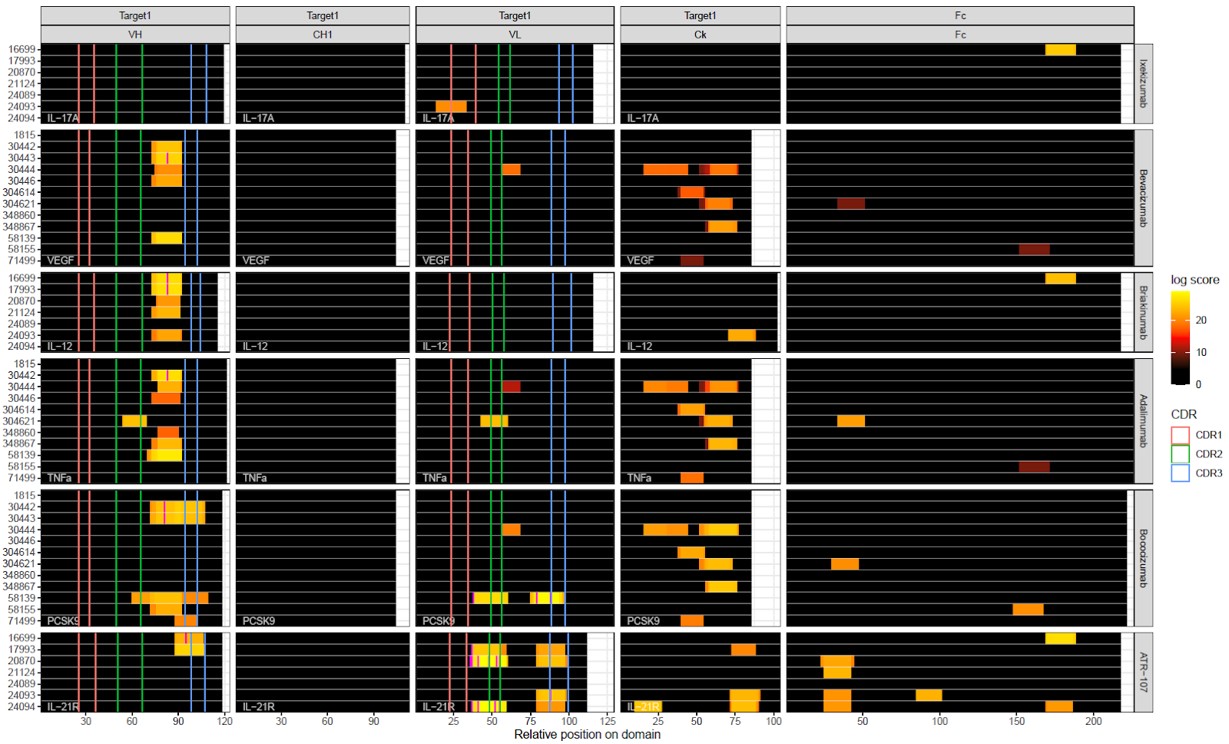

Supplement: Supplementary Figure 3 — Heatmaps depicting the cluster profile of MAPPs-identified peptides. The sequence regions are organized according to the antibody domains (i.e., variable domain of the heavy chain (VH), constant domain of the heavy chain (CH1), variable domain of the light chain (VL), constant region of the kappa-type light chain (Ck), and the fragment crystallizable (Fc) region). Vertical pink, green, and blue lines along the sequence of the VH and VL domains correspond to the position of the complementarity-determining regions (CDRs) 1 to 3. Identified peptide clusters are depicted as colored regions with varying abundances (as a log score) per sequence position, spanning from dark red to yellow. Donor number is denoted on the vertical axis. [file Image3.jpeg]
