# Supplementary material for: Development and characterization of dendritic cell internalization and activation assays contributing to the immunogenicity risk evaluation of biotherapeutics
Source: Front Immunol. 2024 Aug 20;15:1406804. doi: 10.3389/fimmu.2024.1406804 (PMC11368763; doi:10.3389/fimmu.2024.1406804)
Supplement: Supplementary Table 1 — Variance Component Analysis (28) comparing the origins of variance in non-normalized and normalized data. * VC coefficient for “Donor” assigned 0 (shrinkage) VC (Variance Component) denotes extent of the variance emanating from the corresponding variable. Error denotes residual error of the fit. [file Table1.docx]

Supplementary Table 1: Variance Component Analysis [(2)](https://app.readcube.com/library/92e4bd94-af83-4047-ac82-49368190dbce/all?uuid=660996647186651&item_ids=92e4bd94-af83-4047-ac82-49368190dbce:f50edffb-0d0e-4dcd-bc09-fe124c8d21d1) comparing the origins of variance in non-normalized and normalized data.

| Non-normalized data: | | |
| --- | --- | --- |
| Term | VC | % Total |
| **Total** | 0.00443 | 100.00 |
| **Compound** | 0.00215 | 48.51 |
| **Donor** | 0.00139 | 31.32 |
| **Error** | 0.00089 | 20.17 |

| Normalized data: | | |
| --- | --- | --- |
| Term | VC | % Total |
| **Total** | 0.73254 | 100.00 |
| **Compound** | 0.71957 | 98.23 |
| **Donor** | 0* | 0.00 |
| **Error** | 0.01297 | 1.77 |

* VC coefficient for “Donor” assigned 0 (shrinkage)

VC (Variance Component) denotes extent of the variance emanating from the corresponding variable. Error denotes residual error of the fit.

References

1. Champely S. [*pwr: Basic Functions for Power Analysis*](https://app.readcube.com/library/?style=Frontiers%20in%20Immunology+%7B%22language%22:%22en-US%22%7D). (2020). https://CRAN.R-project.org/package=pwr
2. Schuetzenmeister A, Dufey F. [*VCA: Variance Component Analysis*. (2024). https://CRAN.R-project.org/package=VCA](https://app.readcube.com/library/?style=Frontiers%20in%20Immunology+%7B%22language%22:%22en-US%22%7D)
